# Use of Protein Content, Amylose Content, and RVA Parameters to Evaluate the Taste Quality of Rice

**DOI:** 10.3389/fnut.2021.758547

**Published:** 2022-01-13

**Authors:** Shijie Shi, Enting Wang, Chengxuan Li, Mingli Cai, Bo Cheng, Cougui Cao, Yang Jiang

**Affiliations:** ^1^College of Plant Science and Technology, Huazhong Agricultural University, Wuhan, China; ^2^Hubei Collaborative Innovation Center for Grain Industry, Yangtze University, Jingzhou, China

**Keywords:** rice, nitrogen fertilizer, protein, amylose, taste value

## Abstract

Taste quality of rice is the key to its value. However, it is greatly affected by rice types and the environment. It is a complex but necessary factor to accurately evaluate the taste quality of various types of rice in different environments. In this study, 7 different types of rice with different taste values were used as materials, and 12 nitrogen fertilizer treatments were applied to obtain 84 different rice taste values. We used protein content, amylose content, and RVA to evaluate changes in the taste value of rice. Rice with high taste value tended to have higher amylose content, peak viscosity, hold viscosity, final viscosity, and breakdown, as well as lower protein content, pasting temperature, and peak time. Protein and amylose contents affected the taste value of rice by affecting the RVA profiles except for setback. For high and low taste-value rice types, protein content could explain 66.8 and 42.9% of the variation in taste value, respectively. In the case of medium taste-value type, protein content was not enough to evaluate the taste quality of rice. Stickiness could explain 59.6% of the variation in taste value. When the protein content of rice was less than 6.61% or greater than 9.34%, it could be used to reflect the taste quality of rice. When the protein content was in between the two, protein content was not enough to reflect the taste quality of rice. Our results suggested that protein content could better reflect the taste quality change for rice, which provided a theoretical and technical basis for the accurate evaluation of the taste value of various types of rice.

## Introduction

Rice (*Oryza sativa L*.) is the staple food of two-thirds of the world, and rice production has a very important position (1). China is the largest rice producer in the world, and half of the population in China consume rice (2). As living standards improve, the taste quality of rice is becoming more and more important, and people are increasingly fond of high taste-quality rice, and high taste-quality rice also has high economic value in the market (3). The taste quality of rice is affected by many environmental factors, such as variety, temperature, and fertilizer. Only under suitable environmental conditions high taste-quality rice can be produced (4).

The taste quality of rice is a comprehensive evaluation of sensory indicators of rice, such as smell, appearance, palatability, and taste (5). Evaluating the taste quality of rice is often evaluated by manual evaluation or machine evaluation. Different people have different genders, ages, regions, and preferences, etc., which will affect the correct evaluation of rice (6). In recent years, a rice taste analyzer has been used to evaluate the taste quality of rice. The rice taste analyzer uses near-infrared and texture analyzers to comprehensively analyze the appearance and palatability of rice, and then obtain the taste value of rice. Taste value reflects different taste qualities of rice (7). There are also some indirect methods for evaluating the taste quality of rice, such as evaluating the gelatinization properties of rice starch through RVA and then predicting the taste quality of rice. Due to the small sample size, simple use, and high repeatability of the RVA evaluation, they are often used to evaluate the taste quality of rice (8).

The main components of rice are starch and protein. Starch includes amylose and amylopectin. Starch accounts for 80–85% of the chemical composition of rice, while protein accounts for 6–8%. Amylose and protein contents are considered to have an important influence on the taste value of rice (9).

For a long time, people have explored the correlation between RVA and rice taste value (10), but the understanding of protein content, amylose content, and RVA profile difference of different taste-value types of rice is not clear enough. Recently, some scholars have analyzed the taste value of 36 rice varieties in different locations, and they believe that protein content can explain 38.6% of the variation in Indica rice taste value (11). However, the change in the taste value of rice under different nutrient management has not been studied. Nitrogen management has been proved to be one of the most important factors affecting rice taste quality (12, 13). Current research studies have focused on nitrogen fertilizers that reduce the taste quality of rice, and there are only few studies on the evaluation of the taste quality of rice. In this study, we selected 7 rice varieties with large differences in taste value, including conventional Indica rice, hybrid Indica rice, conventional Japonica rice, and Indica-Japonica hybrid rice, and applied 12 nitrogen fertilizer treatments to obtain 84 different taste values. We want to understand the differences in the evaluation of rice taste value by protein content, amylose content, and RVA profiles of different taste value types with nitrogen fertilizer treatments, so as to provide our own suggestions for a more accurate assessment of the taste quality of rice.

## Materials and Methods

In 2019, taste value of 60 various types of rice from 20 origins in China was measured (Supplementary Table S1), and it was found that the taste value of various types of rice ranged from 56 to 91. The 7 rice types used in this study were selected from 60 varieties according to various types, taste value, and grain shape. In 2020, the 7 rice varieties (Tianyuanxiangjing, Yongyou7850, Yongyou4949, Huanghuazhan, Lvyinzhan, Jiafengyou II, and Meixiangzhan II) with different grain shapes and taste values were used for field trials. A field experiment was conducted at a research farm of Jianli County, Hubei Province, China (30°5′ N, 112°56′ E) during the rice-growing season. Plots were arranged in a randomized block pattern with three replicates. The area of each plot was 20 m^2^. Seeds were sown on June 1, 2020, and seedlings were transplanted on July 5, 2020. The rice seedlings were planted in one seeding per hole, and transplanted at an interval of 30^*^13 cm. Superphosphate (50 kg ha^−1^) and potassium chloride (100 kg ha^−1^) were fertilized once before transplanting. Twelve nitrogen fertilizer treatments including 0, 25, 50, 75, 100, 125, 150, 175, 200, 250, 300, and 350 kg N ha^−1^, the total amount of N were applied with the ratio of 5:3 at pre-transplanting and green turning. All the rice varieties were harvested on November 10, 2020. The rice was milled using a rice polisher (Satake, Tokyo, Japan), and then it was used for the next experiment.

### Determination of Protein and Amylase Contents

The rice samples were pulverized (Foss, Hilleroed, Denmark) and passed through a 100-mesh aperture. N concentration of the milled rice was determined using an Elemental analyzer (Elementar, Langenselbold, Germany), and then converted into protein content using a conversion factor (5.95) (14). Amylose content was determined with the iodometric method (15). In short, weigh 0.01 g rice flour in a 15-ml centrifuge tube, add 100 μl of 95% ethanol, and then add 900 μl of 1 mol/L NaOH solution. Place the centrifuge tube in a boiling water bath for 10 min, add 100 μl of 1 mol/L acetic acid after cooling, and then add 200 μl of 0.2% iodine solution; let it stand for 10 min. Epoch Microplate Spectrophotometer (BioTek, Winooski, Vermont, United States) was used to measure the color at 620 nm. Amylose content values were calculated from a standard curve established using mixture solutions of amylose and amylopectin.

### Determination of RVA Profiles

A rapid viscosity analyzer (RVA) (Newport, Warri wood, Australia) was used to determine the RVA profiles of rice. RVA refers to viscosity change of rice starch during heating. The operation process was based on the American Association of Cereal Chemists (AACC) operating procedures (16). About 3g of rice flour was mixed with 25 ml of water, and then put in an aluminum can. The RVA program first heated at 50°C for one minute, then heated to 95°C in 3.75 minutes, and then heated at 95°C for 2.5 min. It was then cooled to 50°C for 3.75 min and held for 1.4 min. Indicators of starch gelatinization included peak viscosity, hold viscosity, final viscosity, breakdown, setback, peak time, and pasting temperature.

### Determination of Taste Value

We used a rice taste analyzer (Satake, Hiroshima, Japan) to determine the taste value of rice. After weighing 30g of rice, we washed it with water within 20 s and placed it in a stainless steel pot to ensure that the ratio of rice to water is 1:1.4 or 1.1.35 (Indica rice was 1:1.4, Japonica rice was 1:1.35, Indica-Japonica rice was 1:1.35). Then we soaked it for 30 min, steamed it in a rice cooker for 40 min, and kept it warm for 10 min. Finally, we placed it at room temperature for 1.5 h to determine the taste value of the rice, including hardness, stickiness and taste value. High taste value often indicates better taste quality.

### Data Analysis

The SPSS 20.0 software (Chicago, IL, United States) and Origin 2021 (Northampton, MA, United States) were used for analysis of variance (ANOVA), cluster analysis, drawing, and correlation analysis. SPSS was also used to perform multiple linear stepwise regression, and the method was stepwise. Significant differences were deemed to occur at *P* < 0.05.

## Results

### Changes in Rice Taste Value With Different Nitrogen Fertilizer Treatments

**Figure 1:**
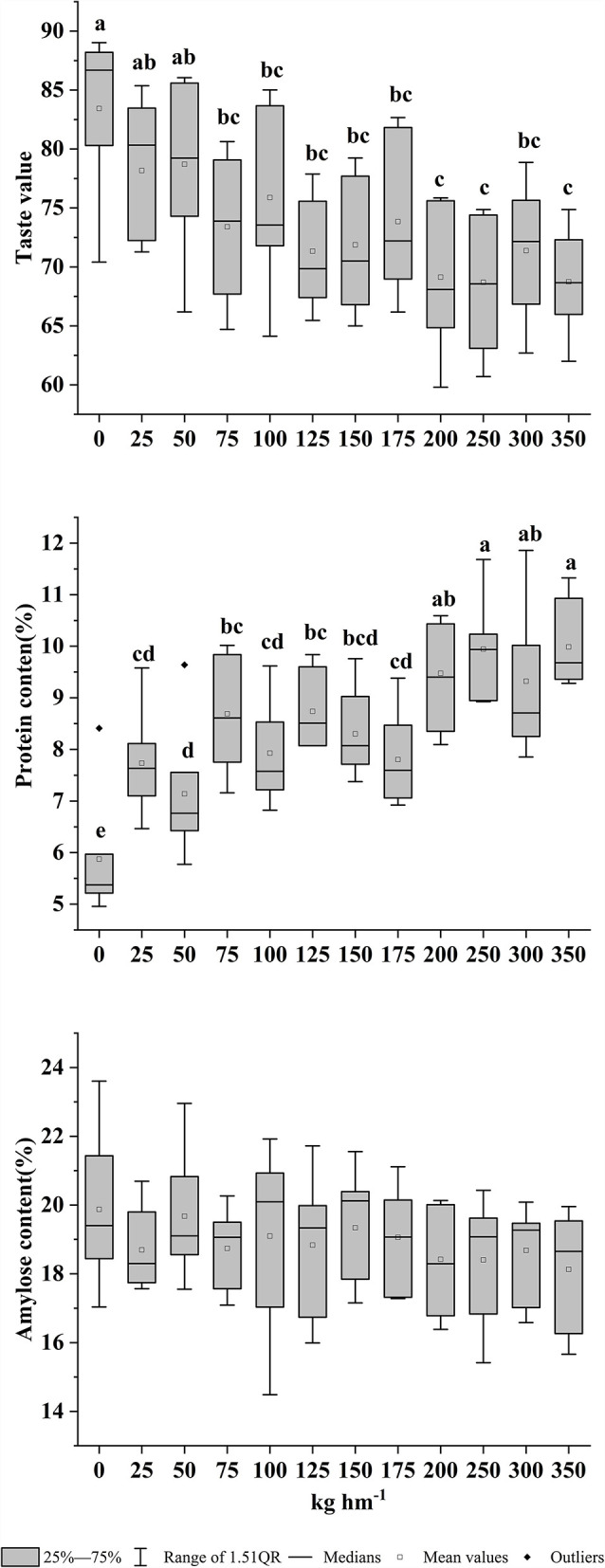
Distribution chart of protein content, amylose content, and taste values with different nitrogen fertilizer treatments. The value of different lowercase letters refers to significant difference at *P* < 0.05.

Under the conditions of nitrogen fertilizer treatment, the protein and amylose contents of rice showed changes, which ultimately led to different rice varieties with different taste values (Figure 1). With different nitrogen fertilizer treatments, the taste value of rice had a wide range [63–89], and nitrogen fertilizer treatments significantly reduced the taste value of rice (*P* < 0.05). With increase in nitrogen fertilizers, the protein content of rice increased significantly (*P* < 0.05). When the nitrogen fertilizer was 250 kg hm^−1^, the protein content reached the highest. With nitrogen fertilizer treatment, amylose content had a downward trend, but the difference was not significant.

### Cluster Analysis of the Taste Value of Various Rice With Different Nitrogen Fertilizer Treatments

**Figure 2:**
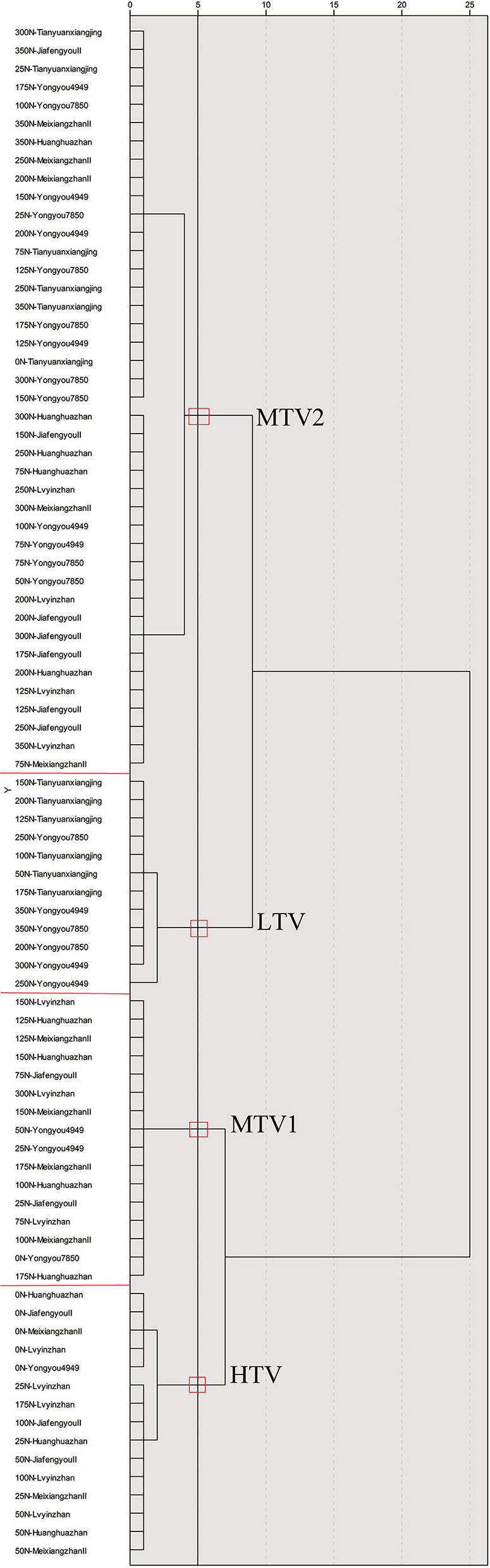
Cluster analysis of taste value of various rice varieties under different nitrogen fertilizer conditions. HTV refers to high taste value type, MTV1 and MTV2 refer to medium taste value type, and LTV refers to low taste value type.

The method of between groups and squared Euclidean distance was used for systematic clustering. Cluster analysis was performed on the taste values of 84 rice under nitrogen fertilizer treatment conditions, and they were divided into high taste value (HTV, from 83 to 89, average = 85.93), medium taste value 1 (MTV1, from 78 to 82, average = 79.75), medium taste value 2 (MTV2, from 68 to 76, average = 72.44), and low taste value (LTV, from 63 to 67, average = 65.42); taste value was ranked from high to low (Figure 2).

### Physical and Chemical Characteristics of the Three Taste Value Types of Rice

**Figure 3:**
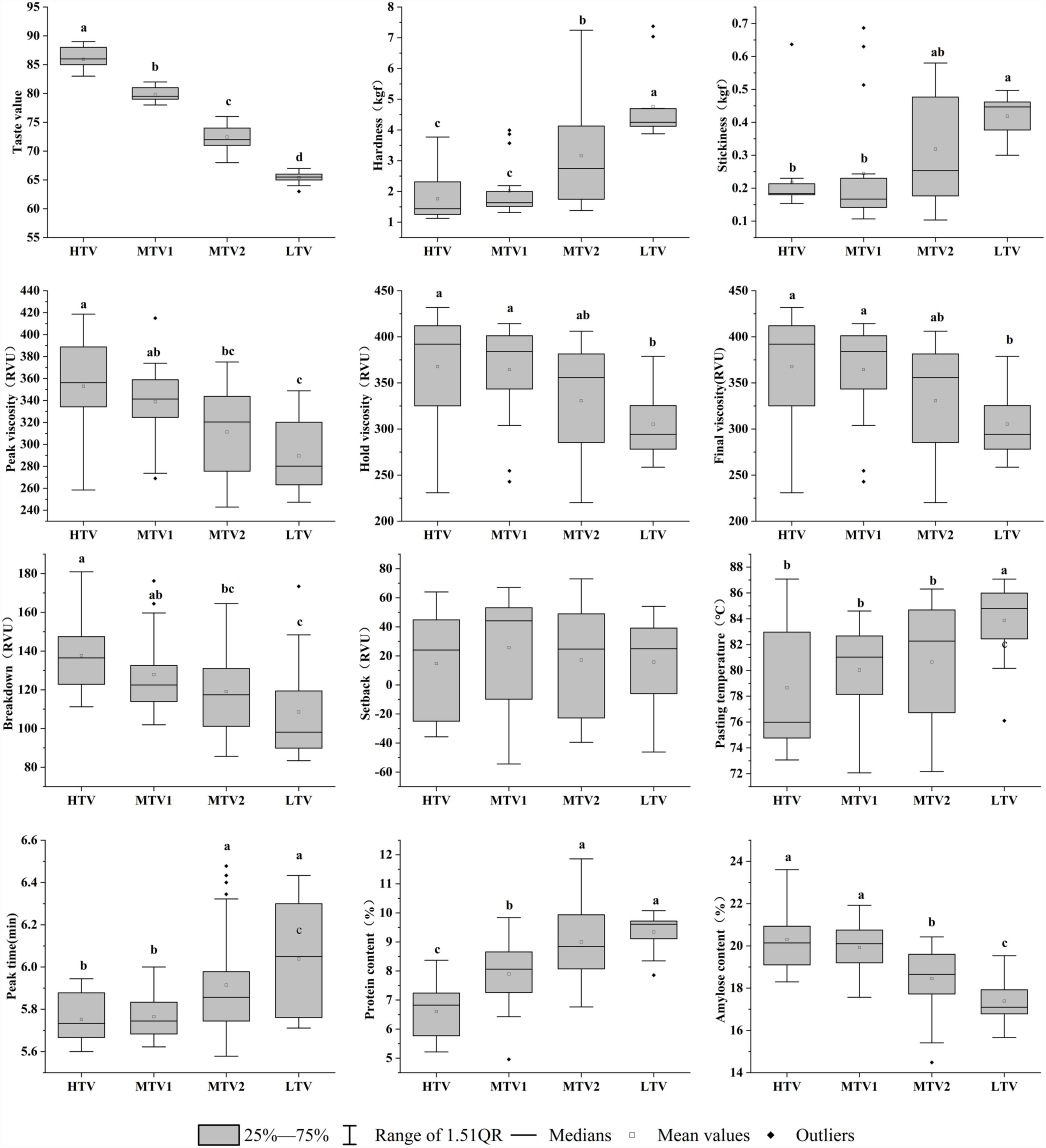
Protein content, amylose content, taste values, and RVA profiles of three different types of rice. The value of different lowercase letters refers to significant difference at *P* < 0.05.

The descriptive statistics of rice with different taste value types are shown in Supplementary Table S3. We could find that setback had the highest coefficient of variation, possibly because the response value had a higher standard deviation, and could be positive and negative values, so the coefficient of variation was larger. In rice of different taste value types, peak time had a lower coefficient of variation, indicating that the peak time of different taste types of rice did not change much. Nitrogen fertilizer had a significant effect on the protein content and taste value of rice, and all the indicators among the rice varieties have significant differences, indicating that protein content may be the most relevant to the taste value of rice. The protein and amylose contents of rice among different taste value types were significantly different (*P* < 0.05) (Figure 3). Compared with the other types, HTV has lower protein and higher amylose contents. The average protein content in HTV was 6.61%, and the average amylose content was 20.31%. LTV had the relatively highest protein and lowest amylose contents. The average protein content in LTV was 9.34%, and the average amylose content was 17.39%. Hence, higher protein and lower amylose contents were associated with LTV.

In this study, it was found that, except for the setback of rice, all RVA profiles were significantly different (*P* < 0.05) (Figure 3). With decrease in rice taste value, the peak viscosity, final viscosity, hold viscosity, and breakdown of rice decreased significantly (*P* < 0.05), and pasting temperature and peak time increased significantly (*P* < 0.05).

The hardness and stickiness of rice are the two most important indicators that affect the taste of rice (17). Cooked rice with a soft and sticky texture is generally preferred by consumers (18). In this study, it was found that the hardness of HTV and MTV1 was significantly lower than that of the other varieties (*P* < 0.05) (Figure 3). There was no significant difference in stickiness among HTV, MTV1, and MTV2, and they were only significantly different from LTV.

### Correlation of Protein Content, Amylose Content, and RVA With Rice Taste Value

**Figure 4:**
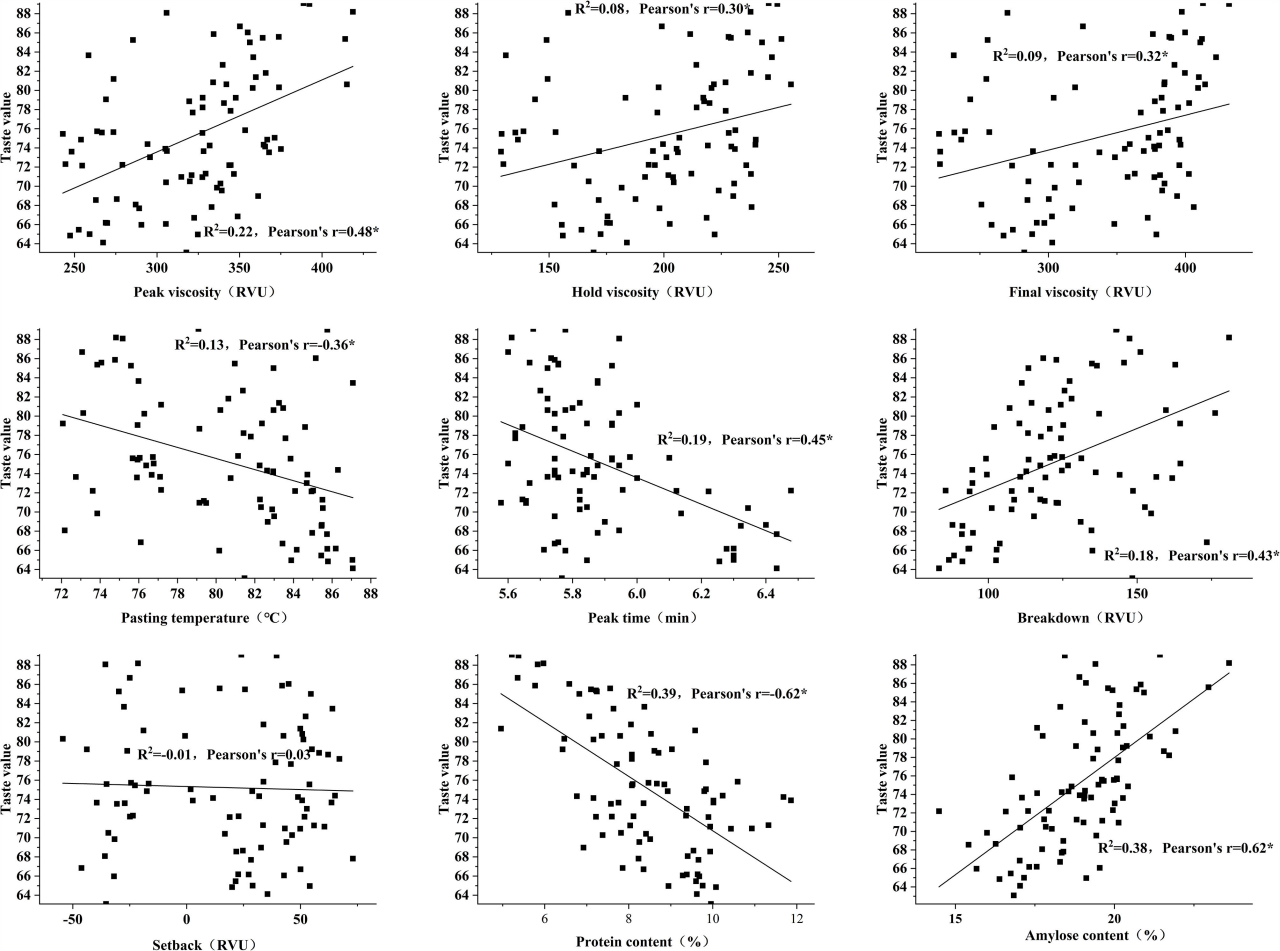
Correlation between protein content, amylase, content and rapid viscosity analyzer (RVA) profiles, and taste values. ^*^Denotes significant difference at 0.05.

In this study, it was found that, except for setback, all the factors have a significant relationship with rice taste value (*P* < 0.05), and that the amylose and protein contents have the highest correlation (r = 0.62 and r = −0.62) (Figure 4). In this study, except for setback, RVA had a significant correlation with protein content (*P* < 0.05) (Table 1). Except for setback and hold viscosity, RVA had a significant relationship with amylose content (*P* < 0.05), indicating that protein and amylose contents affect most of the profiles in RVA to affect the taste value of rice.

**Table 1:** Correlations among protein content, amylose content, and rapid viscosity analyzer (RVA) profiles.

	**Peak viscosity**	**Hold viscosity**	**Breakdown**	**Final viscosity**	**Setback**	**Pasting temperature**	**Peak time**
Protein content	−0.48^*^	−0.29^*^	−0.46^*^	−0.24^*^	0.17	0.28^*^	0.22^*^
Amylose content	0.28^*^	0.21	0.22^*^	0.29^*^	0.15	−0.31^*^	−0.55^*^

* *Denote significant differences at the 0.05*.

### Use of Protein Content, Amylose Content, and RVA Profiles to Evaluate Changes in 84 Rice Taste Values

By multiple stepwise linear regression analysis, taste value was used as the dependent variable, and the protein content, amylose content, and RVA in different taste types were used as the independent variables; then, the insignificant independent variables were eliminated. We found that protein content in the HTV type could explain 66.8% of the change in taste value, and that protein content had the greatest impact on rice taste value (Supplementary Table S4). The stickiness of MTV2 could explain the 59.6% change in taste value. In LTV, the type with the lowest taste value, protein content was still the main factor affecting taste value, and protein content explained the 42.9% change in rice taste value. Therefore, we found that protein content has the most important effect on the taste value among all the influencing factors, and only at the medium taste value types have less influence, at this time stickiness has a better evaluation effect. Too low or too high protein content will determine the taste value of rice. When the protein content was between 6.61 and 9.34%, stickiness has a greater impact on the taste value of rice.

## Discussion

Under the conditions of nitrogen fertilizer treatment, the chemical components in rice changed, protein content increased significantly, and amylose content decreased. Because the amylose content in rice was higher than the protein content (19), the amylose content changed in a larger range, resulting in difference that was not significant. Under the conditions of nitrogen fertilizer treatment, protein content and amylose content show a negative correlation (20). 50N-Lvyinzhan and 250N-Yongyou7850 had a similar amylose content (19.1 and 19.11%, respectively), their protein content was 6.58 and 8.94% respectively, and their final taste values were 86 and 65, respectively. Rice with more than 9% protein content did not taste good, and differences in protein content will cause significant differences in rice taste values (21). It is worth noting that some rice with similar amylose and protein contents have different taste values. The amylose content of 25N-JiafengyouII and 200N-Yongyou4949 was 17.57 and 17.73%, and the protein content was 9.58 and 9.40%. However, the taste values of the two were 81 and 68, respectively. This observation may be due to the different grain types of the two varieties. 25N-JiafengyouIIwas a long-grain variety, while 200N-Yongyou4949 was relatively short in grain length. Previous studies have shown that the morphology of rice grains may affect the water absorption of rice and ultimately affect the taste value (22).

A rapid viscosity analyzer (RVA) is a commonly used tool to evaluate the gelatinization properties of rice starch, peak viscosity reflects the extent of swelling of starch granules, and pasting temperature is the temperature when the starch paste begins to rise (23). Breakdown could evaluate the ease of disintegration of swollen starch granules (24). Setback exhibited the tendency of starch pastes to retrograde, which is an index of starch retrogradation (25). A low setback is indicative of good cooking quality because the rice does not retrograde to become hard upon cooling (26). However, in this study, it was found that there was no significant difference in setback among the different taste value types, which indicated no difference in rice regeneration. Taken together, RVA can well represent the changes in the taste value of rice.

High taste values often indicate lower hardness and higher stickiness (27), but HTV has the lowest hardness and lowest viscosity in this study. Hardness seems to have the greatest negative correlation with the taste of rice (28). We also showed that the effect of hardness on taste value is greater than the effect of stickiness on taste value.

The protein in rice is mainly composed of 4 kinds of protein: albumin, prolamin, globulin, and glutelin (29). Globulin and albumin are mainly distributed in the aleurone layer of rice and are often removed during processing. Therefore, glutelin and prolamin have affected the taste of rice (21). A recent study showed that low gliadin tastes better, which may be the future breeding direction (30). Increase in rice protein content is also related to grain filling rate, and better taste quality can be obtained by coordinating the grain filling (31). Protein plays an important role in the process of rice cooking. Protein can reduce the entry of water molecules into the rice, thereby hindering the gelatinization of starch, and ultimately affecting the taste of rice (32). After the protein is removed, the peak viscosity and breakdown of rice starch increase, and the taste quality of rice is improved (33).

When starch is heated in water, starch granules will gradually expand, and amylose will continue to be leached, and amylopectin will solubilize, resulting in the production of a paste. To a certain extent, the starch pasting behavior determines rice cooking quality and functionality, since starch is the main component of rice (34). Rice starch with low amylose exhibited relatively higher swelling power than high-amylose starch, so low amylose content promotes starch gelatinization (35). The accessions with a high taste quality show an overall trend toward low protein content, low amylose content, high peak viscosity, low pasting temperature, low hardness, high stickiness, and high adhesiveness (36). However, some people use rice with an amylose content of between 13 and 20% and found that there is a positive correlation between amylose content and taste quality. The authors suggest that this may be due to the small difference in amylose content and insufficient sample size (37).

In this study, HTV was accompanied by low protein and high amylose contents, suggesting that protein and amylose contents may have opposite effects on the taste value of rice. Previous studies have considered that protein effects on cooked rice texture were stronger than the amylose effects, that protein content increased and amylose content decreased with increased nitrogen fertilizer levels, and that no effect was observed on taste quality (38).

In summary, nitrogen fertilizer could significantly affect the taste value of rice, and increase in the amount of nitrogen fertilizer application will reduce the taste value of rice. Rice with high taste value under nitrogen fertilizer treatment conditions tended to have lower protein content and higher amylose content. Moreover, rice with high taste value tends to have higher peak viscosity, hold viscosity, final viscosity, breakdown, and lower pasting temperature and peak time. Under nitrogen fertilizer treatments, protein content plays the most important role in affecting the taste of rice. When protein content was less than 6.61% or greater than 9.34%, it could be used to reflect the taste quality of rice. When the protein content was in between the two, stickiness could be a good evaluation of the taste quality of rice. Our results suggested that for most types of rice the protein content could determine the taste.

## Data Availability Statement

The raw data supporting the conclusions of this article will be made available by the authors, without undue reservation.

## Author Contributions

SS: conceptualization, methodology, software, formal analysis, investigation, and writing—original draft. EW: methodology and writing—review and editing. CL, MC, BC, and YJ: writing—review and editing. CC: conceptualization, methodology, investigation, writing—review and editing supervision, and project administration. All authors contributed to the article and approved the submitted version.

## Funding

This study was supported by the Key project of Hongshan Laboratory (No. 2021hszd002), State Key Special Program (Nos. 2018YFD0301304 and 2017YFD0301400), National Natural Science Foundation of China (No. 31701359), and Fundamental Research Funds for the Central Universities (No. 2662019QD049).

## Conflict of Interest

The authors declare that the research was conducted in the absence of any commercial or financial relationships that could be construed as a potential conflict of interest.

## Publisher's Note

All claims expressed in this article are solely those of the authors and do not necessarily represent those of their affiliated organizations, or those of the publisher, the editors and the reviewers. Any product that may be evaluated in this article, or claim that may be made by its manufacturer, is not guaranteed or endorsed by the publisher.

## Supplementary Material

Supplementary Table S1 Taste value of rice of different origins in 2019.

Supplementary Table S2 Protein content, amylose content, RVA characteristics, and taste value of rice of different taste quality types.

Supplementary Table S3 Descriptive statistics of different taste types of rice. Denotes significant difference at 01.

Supplementary Table S4 Multiple regression analysis of protein content, amylose content, and RVA on rice taste value.
